# Pollutants Increase Song Complexity and the Volume of the Brain Area HVC in a Songbird

**DOI:** 10.1371/journal.pone.0001674

**Published:** 2008-02-27

**Authors:** Shai Markman, Stefan Leitner, Clive Catchpole, Sara Barnsley, Carsten T. Müller, David Pascoe, Katherine L. Buchanan

**Affiliations:** 1 School of Biosciences, Cardiff University, Cardiff, United Kingdom; 2 School of Biological Sciences, University of London, Egham, Surrey, United Kingdom; 3 Department of Behavioural Neurobiology, Max Planck Institute for Ornithology, Seewiesen, Germany; University of Cambridge, United Kingdom

## Abstract

Environmental pollutants which alter endocrine function are now known to decrease vertebrate reproductive success. There is considerable evidence for endocrine disruption from aquatic ecosystems, but knowledge is lacking with regard to the interface between terrestrial and aquatic ecosystems. Here, we show for the first time that birds foraging on invertebrates contaminated with environmental pollutants, show marked changes in both brain and behaviour. We found that male European starlings (*Sturnus vulgaris*) exposed to environmentally relevant levels of synthetic and natural estrogen mimics developed longer and more complex songs compared to control males, a sexually selected trait important in attracting females for reproduction. Moreover, females preferred the song of males which had higher pollutant exposure, despite the fact that experimentally dosed males showed reduced immune function. We also show that the key brain area controlling male song complexity (HVC) is significantly enlarged in the contaminated birds. This is the first evidence that environmental pollutants not only affect, but paradoxically enhance a signal of male quality such as song. Our data suggest that female starlings would bias their choice towards exposed males, with possible consequences at the population level. As the starling is a migratory species, our results suggest that transglobal effects of pollutants on terrestrial vertebrate physiology and reproduction could occur in birds.

## Introduction

Numerous examples exist of the detrimental effects of environmental pollutants on the survival or reproductive success of wild organisms e.g.[1]–[3]. In particular, both natural and synthetic endocrine disrupting chemicals (EDCs) act to alter the function of the endocrine system [4], causing gross anatomical changes [5]–[7], as well as changes to behaviour [5] in a range of taxa, including fish, reptiles and amphibians. EDCs' potential to alter physiological function has led to concerns that they could be potent physiological disruptors for wild organisms [7] or, more controversially for humans [8].

According to sexual selection theory [9], male secondary sexual traits have evolved as a result of female preferences and may act as indicators of male quality. Bird song is under strong sexual selection [10] and song production is controlled by discrete neural pathways in the brain which develop and operate under endocrine control of the nervous system [11]. Although the exact roles of testosterone and estrogen in controlling song production are still much debated [12], estrogens are known to be necessary for the masculinisation of the avian song centres in the developing male songbird brain [13]. Furthermore, aromatization of testosterone to estradiol has a neurotrophic effect in male song sparrows (*Melospiza melodia*) and is implicated in controlling the degree of neural plasticity seen in adult songbirds [14]. Many songbird species show seasonal development of their neural song system due to photoperiodic influences on hormone production [15]. This leads to the possibility that neural development in adult birds, which is strongly controlled by the endocrine system may be susceptible to changes in endocrine function.

Natural and synthetic estrogens are known to both occur in concentrated amounts in sewage effluent [16]. As part of sewage treatment processes worldwide, effluent is trickled over filterbeds rich in organic sediment, thereby supporting a complex community of micro and macro-invertebrates [17]. These commonly occurring environments provide an essential foraging environment for a range of wild songbird species, including for one of the most invasive bird species on a global scale, the European starling (*Sturnus vulgaris*), particularly during the winter [18]. The effects of EDC exposure on adult songbird behaviour and physiology are largely unknown, although a recent observational study has documented that neural centres associated with song production may be detrimentally affected by exposure to dichlorodiphenyltrichloroethane (DDT)[19]. This study, which correlated egg levels of a range of DDT metabolites and isomers with neural development in chicks of the American robin (*Turdus migratorius*), found that nestlings with higher total DDT exposure showed reduced forebrain volumes and reduced volume of the *robust nucleus of the archpallium* (RA). DDT is a recognised endocrine disrupter which has complex effects on estrogen receptor activity, but the persistent DDT metabolite p,p′-DDE is a recognised estrogen antagonist and has been shown to inhibit the binding of estradiol both *in vivo* and *in vitro* [20]. Interestingly, in this study the strongest effects on neural development were seen in relation to p,p′-DDE levels in male birds[19].

In the present study, we sought to test the effects of EDC exposure on immune function, song production and neural development in wild birds. Due to the established toxic effects of a range of EDCs on immune function, including changes in antibody production, nitric oxide synthesis, cytokine synthesis, as well as changes to the allergic response [21], we predicted that birds foraging on sewage filterbeds would show immunosuppression. Although EDCs such as DDT can show toxic effects on neural development [19], a range of endocrine disrupters can function as estrogen mimics, potentially having a neurotrophic effect on the development of HVC. Due to the functional association of estrogens with brain masculinisation and neural plasticity [11], [13], [14], we therefore predicted that exposure to EDCs which act as estrogen mimics would, in contrast to exposure to estrogen antagonists[19], cause an increase in both song production and song complexity. We tested these predictions experimentally by exposing wild-caught starlings to ecologically relevant doses of known EDCs and quantifying the effects on immune function and song behaviour. In order to calculate exposure levels of wild starlings, we identified the EDCs present in invertebrate prey and assessed the intake rate of birds observed foraging at these sites. Since we found substantial levels of both natural and synthetic estrogenic compounds [22], we then tested the effects of ecologically-relevant dose levels of either i) 17-β estradiol alone (E2) or ii) a mixture of all the estrogenic compounds found, including E2, on the behaviour and immune function of wild starlings in captivity. Specifically, we predicted that we would see a stepwise decrease in immune function and stepwise increase in song production, song complexity and neural development across the treatment groups, in the order control, E2, mixture treatment.

## Results and Discussion

Wild-caught male starlings (*n* = 36) were randomly allocated to three experimental treatments: 1) control group 2) E2 group or 3) mixture group which received all the known endocrine disrupters identified from field sampling (see Materials and Methods). All dose levels were calculated following field observations of foraging starlings and analysis of invertebrate samples from sewage treatment filterbeds.

**Figure 1:**
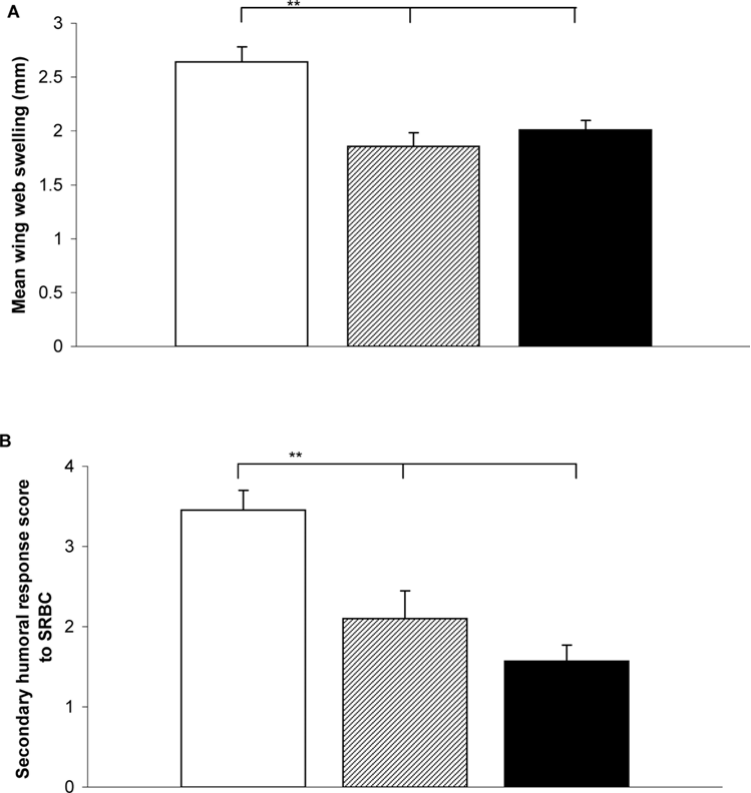
Immune function in male starlings exposed to chemicals. The immune function of male starlings in three treatment groups; control (open bars); E2 dosed (hatched bars); and the chemical mixture dosed (black bars) (a) Cell-mediated immune function was measured as wing web swelling of both wings, 24 hours after injection with phytohaemagglutinin (PHA). Treatment had a significant effect on cell-mediated immune function (ANOVA, *F*_2, 32_ = 12.16, *P*<0.001). Bonferroni pairwise comparison post-hoc tests showed that the immune function of males in both chemically dosed groups (E2 or mixture) was significantly lower than that of the control males (E2 versus control *P*<0.001, mixture versus control *P* = 0.001) but there was no significant difference between males in the E2 and mixture groups (*P*>0.05). (b) The secondary humoral response following an intraperitoneal injection of sheep red blood cells (SRBC). Treatment had a significant effect on the secondary humoral response to SRBC (ANOVA, *F*_2, 32_ = 10.98, *P*<0.001). Bonferroni pairwise comparison post-hoc tests showed that the mean response of the males in both dosed groups (E2 or mixture), was significantly lower than the mean of the control males (E2 versus control *P*<0.001, mixture versus control *P* = 0.001), but there was no significant difference between the E2 treated and the mixture treated males (*P*>0.05). Graphs show means+s.e.m. ** indicates *P*<0.001.

EDC exposure significantly reduced both cell-mediated immune function (Fig. 1a) and the humoral immune response of male starlings (Fig 1b). Treatment did not have an effect on body mass (ANOVA, *F*_2, 32_ = 0.334, *P* = 0.718) nor on haematocrit (% packed red blood cell volume) (ANOVA, *F_2, 30_* = 1.338, *P* = 0.278) or testosterone titre (ANOVA *F*_2, 32_ = 0.66, *P* = 0.524), as measured at the end of the experimental period.

**Figure 2:**
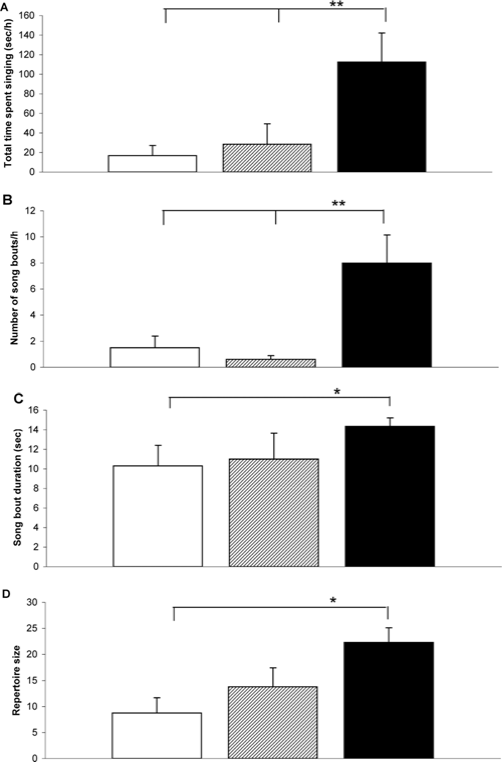
Song production in male starlings exposed to chemicals. The song production of male starlings in three treatment groups: control (open bars); E2 dosed (hatched bars); and the chemical mixture dosed (black bars) (a) Total time spent singing (sec/h). (b) Number of song bouts per hour. (c) Song bout duration (s) d) Repertoire size. Graphs show means+s.e.m. There was a significant effect of the experimental manipulation on the time spent singing between the treatment groups (ANOVA, *F_2, 24_* = 6.15, *P* = 0.007). Bonferroni pairwise comparison post-hoc tests showed that the males that received the mixture of chemicals spent significantly longer singing than the control males (*P* = 0.009) and the E2 group (*P* = 0.028). There was a significant effect of treatment on the number of song bouts sung by the males (ANOVA, *F_2, 23_* = 9.16, *P* = 0.001). Males in the mixture treatment group sang more song bouts than the control males (*P* = 0.004) and the E2 males (*P* = 0.002). Mean song bout duration was significantly longer for males in the mixture treatment group compared to the control males (ANOVA, *F_1, 11_* = 5.842, *P* = 0.034). Finally, there was a significant effect of the experimental manipulation on the repertoire size of male starlings (ANOVA *F_2, 16_* = 4.39, *P* = 0.030). The males in the mixture group had significantly greater repertoire size than males in the control group (Bonferroni pairwise comparison post-hoc tests *P* = 0.042). * = *P*<0.05; ** = *P*<0.01.

**Figure 3:**
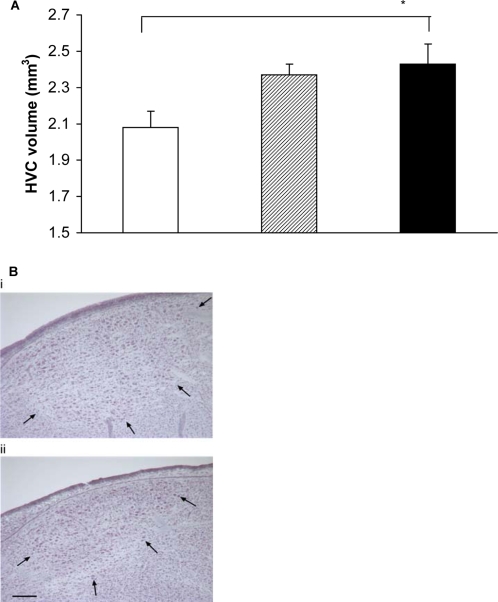
HVC size in male starlings exposed to chemicals. a) HVC volume (mean+s.e.m.) in the three treatment groups; control (open bars); E2 dosed (hatched bars); and the chemical mixture dosed (black bars) (ANOVA, *F*_2, 32_ = 4.46, *P* = 0.019). HVC volume of the males in the mixture group was significantly larger than in males in the control group (Bonferroni pairwise comparison post-hoc tests *P* = 0.032), but there were no significant differences in the HVC volume between males in the E2 and control groups (*P*>0.05) or between males in the E2 and mixture groups (*P*>0.05) * = *P*<0.05. b) Photomicrograph of an HVC from (i) a chemical mixture treated male and (ii) a control male. Arrows indicate the borders of HVC. Scale bar = 200 µm.

Treatment had a significant effect on the song output of the male starlings (Fig. 2). Males in the group which received the mixture of chemicals spent more time singing, sang more song bouts, sang longer song bouts and had a larger repertoire size, a measure of song complexity, than males in the control group. The mechanism for this effect is clear as when examining the underlying neurobiology. There was a significant effect of treatment on HVC volume, the principal nucleus in the songbird brain associated with the production of complex songs [12], [23], such that the HVC volume of the males in the mixture group was significantly larger than in males in the control group (Fig. 3a, b). There were no significant differences in the HVC volume between males in the E2 and control groups or between males in the E2 and mixture groups (Fig. 3a).

**Figure 4:**
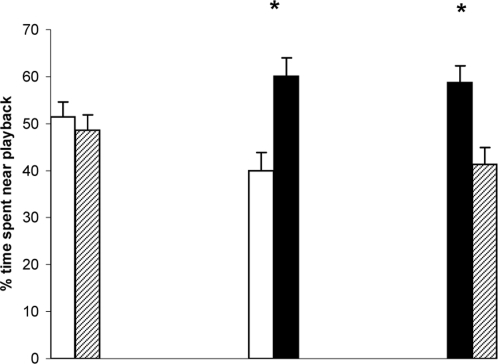
Song preferences in female starlings. The percentage of time spent by females on the perch adjacent to song playback from male starlings in the three treatment groups; control (open bars); E2 dosed (hatched bars); and chemical mixture dosed (black bars). Playback from the mixture group was preferred over playback from E2 dosed males (*t*_10_ = 2.42, *P* = 0.035); Playback from the mixture group was preferred over song from control males (*t*_9_ = 2.57, *P* = 0.029). There was no significant preference between control and E2 dosed playback (*P*>0.05); Graphs show mean+s.e.m. * = *P*<0.05.

Finally, consistent with the changes in repertoire size and underlying neural structure, in mate choice preference tests female starlings showed a significant preference for song playback from males dosed with the mixture of chemicals in comparison to control males (Fig. 4). In addition, song from males exposed to the mixture of chemicals was preferred over song from the E2 dosed males, although no difference was found between the preferences for song from E2 dosed or control males.

To the best of our knowledge, our study provides the first experimental test of the effects of ecologically relevant dose levels of endocrine disrupters on avian neural development and behaviour. Our dose levels have been carefully determined following field observations and sampling [22] and assume that starlings in winter take approximately half of their food intake from the sewage filterbeds. The fact that EDC exposure can have detrimental effects on immune function [21] is supported by the consistent immunosuppression across the treated groups. The higher EDC dose of the mixture group did not cause further increased levels of immunosuppression, above that of the E2 group, although there are a number of potential interpretations of this result.

Steroid hormones influence the initial sexual differentiation of the songbird brain [11] and 17-β estradiol is specifically known to affect the plasticity of the avian brain [14], [24], suggesting that estrogen mimics and natural estrogens can directly influence seasonal development of HVC. Males that received the mixture of EDCs showed both increased song output and increased song complexity, almost certainly due to changes in the size of the HVC. We saw no such effects within the group dosed solely with E2. This could be due to the fact that the physiological response to EDC exposure is dose-dependent, such that the higher total dose of EDCs in the mixture group produced effects that E2 alone would not. Alternatively, these effects could be due to the combination, or even a subset, of the pollutants administered in the mixture treatment. Within the brain of the songbird, testosterone is converted into estrogen, which is then released into the blood stream at physiologically significant levels [25]. Paradoxically, in birds where male is the default sex, estrogens are known to be both necessary for the feminisation of the sexual organs during early development, and also for the masculinisation of the avian song centres in the brain [13]. Song complexity determines male attractiveness in many songbird species [10], and has been shown intraspecifically to correlate with the volume of the HVC [23]. Within the cerebral song system pathways only the HVC has estrogen receptors [12], [26] suggesting this region is likely to be one of the most susceptible to the effects of EDCs. Our study has demonstrated the vulnerability of the HVC to disruption by estrogen mimics. In addition, our results also highlight the continued plasticity of the adult songbird brain.

From an ultimate, evolutionary perspective our results suggest that exposure to endocrine disrupters may alter the selective forces acting on songbird populations. It is established that female starlings show active preferences for males which have greater song output and larger repertoire sizes [27]. Our results show that females prefer the song output from males exposed to the complete mixture of endocrine disrupters, despite the fact that such males are immunosuppressed. If female starlings bias their reproductive investment towards males in poor physiological condition then hatching and/or fledging rates could decline with detrimental consequences at the population level.

Our findings document for the first time that invertebrates living on sewage filterbeds take up a range of environmental pollutants. The levels of these chemicals in aqueous sewage effluent leaving the percolating sewage filterbeds in UK have been found to vary: e.g. E2 50 ng/L[16]–100 ng/L [28] or bisphenol A 500 ng/L [28]. Although liquid and solid samples are not analogous, the concentrations of both E2 and bisphenol A identified by our study in 1 g of earthworms therefore greatly exceed (up to 1000 fold) [22] those previously reported in 1 ml of sewage treatment effluent. Our study therefore highlights the potential for such pollutants to have detrimental physiological effects at various trophic levels.

Birds are transglobal vectors for disease and our results highlight the potential for them to demonstrate intercontinental effects of pollution exposure. As the starling is a migratory bird species, our findings may suggest that pollutant exposure on the wintering grounds could affect reproductive success at the breeding sites. Starling populations in the UK have suffered a 50% decrease in the last forty years and consequently the starling is listed as a bird of high conservation concern [29]. Many issues contribute to this decline [29], but reduced reproductive success as a result of EDC exposure may be a factor that has yet to be recognised. Our study has shown that ecologically-relevant levels of EDC intake affect immune function, neural development and behaviour in male starlings and may therefore contribute to their population decline. Further work is needed to quantify the importance of these effects in wild bird populations.

## Materials and Methods

### Quantification of contamination levels

We observed starlings foraging in the winter of 2003/4 at 20 sewage treatment works in the south west UK, and their prey species were identified. We collected and analyzed duplicate 10 g samples of the earthworm *Eisenia fetida*, which was the prey item observed to be taken at the greatest biomass. The EDC content of the collected earthworm tissue was quantified using gel permeation chromatography and GC-MS [22]. The mean±s.e of each chemical in the earthworm samples across five sewage filterbed sites were: 9.85±6.7 ng/g of 17 β estradiol (E2), 6.2±2.19 ng/g dibutylphthalate, 26±12.6 ng/g dioctylphthalate and 4.28±2.6 ng/g bisphenol A. We found that *E. fetida* from garden soil contained significantly lower levels of these chemicals excluding E2 which was only found in the earthworms from the sewage treatment sites [22]. Starlings were observed to take in single *E. fetida* (mean mass 0.3 g) at a rate of 1/min, with a mean patch residence time of 16 mins/hr observation and the intake rate was constant with increasing food patch residence time (*P*>0.05). We therefore calculated from our observations that the individual starlings in our study take in on average 14.4 g/day wet weight of invertebrates from the sewage treatment filterbeds. As the daily food intake of invertebrates (wet weight) for adult starlings is approximately 30 g/day [18], intake from filterbeds represents 48% of their daily food intake during the winter months (100.8 g wet weight/week). The daily dose levels used in the captive experiment were based on the chemical content of the filterbed samples and this intake calculation.

### Dosing and physiological responses of captive birds

One year old starlings were allocated to three treatment groups: 1) control group: each bird received daily one mealworm *Tenebrio molitor* with 10 µl of peanut oil, injected into the body cavity as the carrier substance, 2) E2 group: each bird received a mealworm with 200 ng 17- β estradiol (E2) in 10 µl of peanut oil, or 3) mixture group: each bird received a mealworm with 200 ng E2, 520 ng dioctylphthalate, 80 ng bisphenol A, and 120 ng dibutylphthalate dissolved in 10 µl of peanut oil. All the birds were caught as juveniles and housed for one year in outdoor aviaries prior to the start of the experiment. During the experiment the birds were housed in single-sex trios (1 from each treatment group) in outdoor aviaries each measuring 2 m×1 m×1 m and maintained in the same groups throughout the experiment. Birds were dosed 5 days per week from October 2004 until April 2005, to mimic their foraging period on sewage filter beds. All the starlings were maintained on an *ad lib* diet of an insect paté (Orlux™) and had constant access to water and one nestbox per bird. At the end of the experiment (April 2005), the birds were also weighed and blood sampled for haematocrit levels and testosterone levels. The cell-mediated immune response of the birds was tested in March 2005, by using an injection of phytohaemagglutinin (PHA) into both wings webs [30]. The thickness of both wing webs was measured (mean 3 measurements) at the same location of the wing before injection and 24 hours after injection, using callipers (Moore and Wright™; to 0.1 mm). PHA (Sigma L-8754) in phosphate buffered saline (PBS; 0.45 µg in 50 µl [30], [31] was injected into both wings webs of each bird. The mean response of both wings was calculated and used in all analyses. A control injection of PBS alone, to control for any injection trauma was not carried out, as this has been shown to be unnecessary [32]. After more than 24 hours post-injection, the swelling subsided. The humoral response of the birds was tested in April 2005 using intraperitoneal injection of sheep red blood cells (SRBC) [33]. A control blood sample was drawn before the start of the test. SRBC in Alsever's solution (TCS Microbiol Ltd, Claydon, UK) washed and resuspended 1× PBS to form a 2% solution. 500ul was injected twice intraperitoneally 14 days apart and blood samples were drawn to test for the primary and secondary humoral response. Plasma samples were heat-treated at 56°C for 30 minutes and stored at −20°C for three weeks before testing using a standard haemagglutination test[33].

### Song analysis

In March-April 2005 the song output of individual male starlings was recorded as follows: On day 1 a male was moved into a separate outdoor cage with a novel female. Each male was housed with a different female to avoid pseudoreplication. The song output of the male was recorded twice for 3 hours: once in the afternoon of day 1 and once on the morning of day 2. The recordings were made using a Marantz solid state recorder PMD 670 and a Sennheiser K6 microphone body, with a Sennheiser (MKE 2-60 Gold C) sub-minature microphone attachment, mounted ontop of the nestbox. We calculated the following measures: (i) the total amount of time spent singing; (ii) the number of song bouts; (iii) the duration of each song bout and iv) repertoire size. Song bouts were defined as continuous song and were separated from each other by at least 1 s [27]. Repertoire size was estimated from a cumulative plot of the novel phrase types appearing in 20 song bouts [27]. These measures were averaged over both recording periods. Birds that did not sing during either recording attempt were not included in the song analysis.

### Testosterone analysis

Testosterone concentrations were estimated from plasma samples in 2 assays using anti-testosterone antiserum (code 8680-6004, Biogenesis, U.K.) and [^125^I]-testosterone label (code 07-189126, ICN, U.K.) [34]. The mean 50% binding for the assays was 0.355 ng/ml. Samples were run in either duplicate 10ul or 20ul samples and the detection limits were of 0.01 ng/ml or 0.02 ng/ml respectively. The interassay CV was 10.4%.

### Neural analysis

Male starlings were killed by decapitation on 22^nd^ April 2005 and their brains were removed immediately by dissecting them out of the skull. Brains were frozen over liquid nitrogen and stored at −80°C until analysis. Brains were cut on a cryostat (Leica) into 30 µm sagittal sections. Sections were mounted onto Superfrost Plus slides (Menzel Gläser, Germany) in four different series. One series was Nissl-stained with thionin and cover slipped. Slides were analysed under bright-field illumination with a microscope (Leitz Aristoplan). For area measurements, brain regions were video-digitised on a PC equipped with an image analysis system (Meta Morph, Visitron, Germany) and measured by the built-in measurement tools. Volumes were calculated as the sum of the area sizes multiplied by section interval and section thickness.

Throughout, all statistical analysis was conducted using Systat v 10. As the males were held in trios, trio group was entered as a covariate in all models, but not found to be significant in any case (*P*>0.05).

### Song preference

Wild-caught female starlings (*n* = 11) were placed in a long aviary (810×180×200 cm) with a perch 20 cm away from each of the speakers at each end. A Sony SRS-A37 speaker was hidden behind a cloth at each end of the arena. The speakers were connected to a Sony Walkman portable compact disk player operated by the experimenter.

Five males were used from each treatment group to provide the song stimuli and were randomly paired in the combinations of playback from the different treatment groups. Two different song files were created using Avisoft-SASLAB Pro for each male, each containing three song bouts, for a total clip length of 30 s. These songs were matched for amplitude and used to create 5 minutes song loops, which were then recorded 6 times to create 30 minutes of song stimulus. The playback from a particular male was experienced by either 2 or 3 females and averaged across females. Choice stimuli tests were counterbalanced for each song pair type and side of presentation (right or left speaker) in the testing apparatus and playback was simultaneous at each end of the aviary.

We tested each female with three comparisons of male song stimuli in succession: control versus males treated with the mixture of chemicals; control versus E2 treated males; and E2 treated versus mixture of chemical males. Each test consisted of two playback blocks. In each block the song playback was played for 5 minutes prior to data gathering. The song stimuli were played for 30 minutes. The amount of time that the female spent on the perches within 20 cm of the speaker was recorded. In the second block the protocol was repeated but the song playback was reversed to control for any side biases. Data were averaged over blocks 1 and 2. The playbacks of the treatment comparisons were carried out sequentially and the order of the pairwise song stimuli choice test was randomly assigned to each female.
